# 

**Published:** 1860-07

**Authors:** 


Published Monthly, at $3 per Annum in Advance.

### ATLANTA MEDICAL AND SURGICAL JOURNAL.

EDITED BY

**JOSEPH P. LOGAN, M. D.,**

Professor of Physiology and Diseases of Women and Children in the Atlanta Medical College.

AND

**W. F. WESTMORELAND, M. D.,**

Professor of the Principles and Practice of Surgery in the Atlanta Medical College.

**Pax et scientia, sed veritas sine timore.**

Vol. V. JULY, 1860. No. XI.

ATLANTA, GA:

ATLANTA INTELLIGENCER PRINT.

1860.

Each Number contains Sixty-Four Pages.

